# Expected years of life lost through road traffic injuries in Mexico

**DOI:** 10.1080/16549716.2017.1360629

**Published:** 2017-08-18

**Authors:** Efrén Murillo-Zamora, Oliver Mendoza-Cano, Benjamín Trujillo-Hernández, José Guzmán-Esquivel, Alfredo Medina-González, Miguel Huerta, Ramón Alberto Sánchez-Piña, Agustin Lugo-Radillo

**Affiliations:** ^a^ Coordinación de Vigilancia Epidemiológica, Jefatura de Servicios de Prestaciones Médicas, Instituto Mexicano del Seguro Social, Colima, México; ^b^ Center for Health and the Global Environment, Harvard T.H. Chan School of Public Health, Boston, MA, USA; ^c^ Facultad de Ingeniería Civil, Universidad de Colima, Colima, México; ^d^ CONACYT- Facultad de Medicina y Cirugía, Universidad Autónoma Benito Juárez de Oaxaca, Oaxaca, México; ^e^ Facultad de Medicina, Universidad de Colima, Colima, México; ^f^ Unidad de Investigación en Epidemiología Clínica, Instituto Mexicano del Seguro Social, Colima, México; ^g^ Coordinación de Planeación y Enlace Institucional, Jefatura de Servicios de Prestaciones Médicas, Instituto Mexicano del Seguro Social, Colima, México; ^h^ Centro Universitario de Investigaciones Biomédicas, Universidad de Colima, Colima, México

**Keywords:** Traffic accidents, premature mortality, burden of illness, Mexico, health Policy

## Abstract

**Background**: Road traffic injuries (RTIs) are a leading cause of premature mortality, mainly in low- and middle-income countries

**Objective**: To estimate the 2014 burden of RTIs in Mexico calculating years of life lost (YLL) and age-standardized YLL rates (ASYLL), and to evaluate sex, age, and region-related differences in premature mortality.

**Methods**: Mortality data were obtained from the National Institute of Statistics and Geography and 14,637 deaths of individuals 15 years of age and older were analyzed. The YLL and ASYLL were computed.

**Results**: The overall burden of RTIs was 332,922 YLL and 82.4% of the deaths occurred in males. Males from 25 to 34 years of age and females from 15 to 24 years of age showed the highest age-adjusted YLL rates (933 and 158 YLL per 100,000 inhabitants, respectively). The national ASYLL rate was 416 per 100,000 inhabitants and the highest state-stratified mortality rates were observed in Tabasco (851), Sinaloa (709), Durango (656), Zacatecas (642), and Baja California Sur (570).

**Conclusions**: RTIs contributed to the premature mortality rate in the study population. Our findings may be useful from a health policy perspective for designing and prioritizing interventions focused on the prevention of premature loss of life.

## Background

Road traffic injuries (RTIs) are a leading cause of premature mortality, mainly in low- and middle-income countries [1]. An increase in the related burden has been observed worldwide and pedestrians, cyclists, and two-wheeled motorcycle riders are the most vulnerable populations that contribute to the observed mortality [2,3]. In Mexico, the overall RTI mortality has decreased since 2009; however, an increase in fatal motorcycle mortality has been observed and RTIs are still a main cause of death [4–6].

Traditional mortality rates have been used to evaluate the RTI burden in Mexico, which fail to highlight the premature deaths [7]. In 2015, the country adopted the United Nations Sustainable Development Goal on health (SDG3) and its aim is a 40% reduction of premature deaths by the year 2030. RTIs are one of the targeted causes of premature death [8]. A cost-effectiveness approach is needed to reach the proposed objective and the estimation of standard expected years of life lost (YLL) may be feasible and beneficial.

The YLL measure was developed by the Global Burden of Disease (GBD) study and is a useful analytical tool, from an economic and health policy perspective, for measuring preventable loss of life. The disability-adjusted life year (DALY) is calculated as the sum of the YLL and the years lived with disability (YLD). This health measure incorporates strategies (i.e. time-based discounting) used in cost-effectiveness analyses. In addition, the age-standardized YLL (ASYLL) has been used to compare goals across populations [9] and is a valid measure for identifying demographic or regional subgroups (i.e. the states of a country) with the highest premature mortality rate [10].

To the best of our knowledge, there are no published studies evaluating regional premature mortality due to traffic accidents in Mexico. This approach may be useful to identify high risk subgroups where particular interventions focused on the prevention of premature loss of life may be beneficial. The aim of this study was to estimate the burden of RTIs through YLL and ASYLL in Mexican individuals of 15 years of age and older at death in 2014. In addition, sex, age, and region-related differences were evaluated.

## Methods

The YLL for RTIs were calculated following the methods described by the GBD study [11]. First, sex and age-stratified (15–24, 25–34, 35–44, 45–64 and ≥65 years of age) mortality data were obtained from the National Institute of Statistics and Geography of Mexico [12]. The following underlying causes of death (International Classification of Diseases 10th revision, ICD-10) were included: V09.2, V09.3, V12–V14, V19.4–V19.9, V20–V28, V29.4–V29.9, V30–V39, V40–V49,V50–V59, V60–V69,V70–V79,V80.3–V80.5, V81.1, V82.1, V83.0–V83.4,V84.0–V86.4, V87.0–V87.8, V89.2, and V89.9. These events are clustered using code E49B (Mexican List of Causes of Death) for statistical purposes [13].

Second, the YLL measure was computed for males and females by multiplying the number of RTI deaths by the number of years of expected remaining life at the respective age interval according to the 2013 Tables of Life (Global Health Observatory) of Mexico [14]. The total population was obtained from the 2010 National Census of Population and Housing [15]. The average age of death, per age interval, was calculated using national data from the Statistical and Epidemiological Death Registration System [16]. The following parameters were fixed: discount rate (*r*) = 0.03, age-weighting (*β*) = 0.04, adjustment constant for age-weights (*C*) = 0.1658, and age-weighting modulation (*K*) = 0. Templates (Microsoft® Excel®) from the GBD study were used to compute the YLL and the summary statistics were estimated using Stata® MP 13.0 (StataCorp LP). Finally, the ASYLL rates per 100,000 inhabitants were estimated using the World Standard Population (2000–2025).

The Local Health Research and Ethics Committee of the Mexican Institute of Social Security approved the present study.

## Results

The number of RTI-associated deaths in 2014 was 11,944 in males and 2693 in females (Table 1), representing 2.5% of all deaths occurring in Mexico within the same period. The unadjusted mortality rates per 100,000 inhabitants were 31.7 in males (ranging from 18.4 to 66.0) and 19.0 in females (ranging from 4.2 to 12.1).

**Table 1.** Deaths by road traffic injuries, Mexico 2014.

	Males			Females			Overall		
State	*n*	%	Rate	*n*	%	Rate	*n*	%	Rate
Aguascalientes	170	(5.9)	44.2	44	(1.9)	10.4	214	(4.1)	26.5
Baja California	250	(2.7)	22.4	89	(1.5)	8.1	339	(2.2)	15.3
Baja California Sur	91	(5.8)	39.6	25	(2.4)	11.4	116	(4.4)	25.8
Campeche	100	(4.5)	35.2	29	(1.7)	9.8	129	(3.3)	22.3
Coahuila	366	(4.3)	38.9	92	(1.4)	9.5	458	(3.0)	23.9
Colima	90	(4.3)	39.4	16	(1.1)	6.8	106	(3.0)	22.8
Chiapas	455	(3.8)	30.5	83	(0.8)	5.2	538	(2.4)	17.4
Chihuahua	364	(3.1)	31.9	123	(1.4)	10.4	487	(2.4)	21.0
Mexico City	580	(2.0)	18.4	209	(0.8)	5.9	789	(1.4)	11.7
Durango	265	(5.5)	49.3	66	(1.9)	11.6	331	(4.0)	29.9
Guanajuato	642	(4.2)	36.5	144	(1.2)	7.2	786	(2.9)	21.0
Guerrero	309	(3.4)	29.0	89	(1.3)	7.5	398	(2.5)	17.7
Hidalgo	276	(3.8)	31.6	85	(1.4)	8.7	361	(2.7)	19.5
Jalisco	875	(4.0)	35.4	209	(1.2)	7.9	1084	(2.7)	21.1
Mexico	1557	(4.0)	30.5	359	(1.1)	6.5	1916	(2.7)	18.0
Michoacan	370	(2.8)	26.1	89	(0.9)	5.6	459	(1.9)	15.3
Morelos	129	(2.4)	21.6	40	(0.9)	6.0	169	(1.7)	13.4
Nayarit	146	(4.5)	38.8	36	(1.5)	9.3	182	(3.2)	23.9
Nuevo León	400	(3.1)	24.3	123	(1.2)	7.3	523	(2.2)	15.7
Oaxaca	354	(3.1)	29.3	86	(0.9)	6.2	440	(2.1)	17.0
Puebla	541	(3.2)	29.6	116	(0.8)	5.5	657	(2.1)	16.7
Queretaro	241	(5.2)	39.9	56	(1.4)	8.4	297	(3.5)	23.4
Quintana Roo	131	(4.8)	27.9	21	(1.1)	4.6	152	(3.3)	16.4
San Luis Potosi	359	(4.7)	42.2	83	(1.4)	9.0	442	(3.2)	24.9
Sinaloa	533	(6.3)	55.0	98	(1.7)	9.8	631	(4.5)	32.0
Sonora	371	(4.1)	39.6	92	(1.5)	9.8	463	(3.1)	24.7
Tabasco	494	(7.8)	66.0	96	(2.0)	12.1	590	(5.3)	38.2
Tamaulipas	383	(3.9)	34.6	123	(1.6)	10.6	506	(2.9)	22.3
Tlaxcala	127	(4.4)	33.3	26	(1.0)	6.1	153	(2.8)	19.0
Veracruz	515	(2.0)	20.1	121	(0.6)	4.2	636	(1.4)	11.7
Yucatan	214	(3.4)	31.2	46	(0.9)	6.4	260	(2.3)	18.5
Zacatecas	246	(5.5)	50.3	49	(1.3)	9.2	295	(3.6)	28.9
**Overall**	**11,944**	**(3.6)**	**31.7**	**2963**	**(1.1)**	**7.3**	**14,907**	**(2.5)**	**19.0**

The absolute frequencies (*n*), proportions (%) from the total number of registered deaths, and unadjusted mortality rates per 100,000 inhabitants are presented.

Data source: Main causes of mortality by place of residence, age and sex, 2014; National Institute of Statistics and Geography.

Table 2 shows the YLL by sex, age group, and state of residence. The YLL was higher in males in all age groups. The overall YLL in males was 274,451 and the highest age-adjusted rate (933 per 100,000 inhabitants) was observed in individuals from 25 to 34 years of age at death. The YLL in females was 58,470 and the highest rates were observed in young females (15–24 years of age, 158 YLL per 100,000 inhabitants).

**Table 2.** Years of life lost by road traffic injuries and age-adjusted rates per 100,000 inhabitants, Mexico 2014.

	15–24 yr.		25–34 yr.		35–44 yr.		45–64 yr.		≥65 yr.	
State	YLL	ASYLL	YLL	ASYLL	YLL	ASYLL	YLL	ASYLL	YLL	ASYLL
**Males**										
Aguascalientes	1161	1036	772	892	829	1106	811	971	211	769
Baja California	1501	506	1252	470	1463	594	1444	603	144	219
Baja California Sur	481	802	746	1278	341	703	455	915	76	571
Campeche	651	825	746	1173	463	844	455	717	59	252
Coahuila	2237	902	2476	1177	1536	809	1464	671	482	654
Colima	680	1114	612	1211	268	610	435	817	85	438
Chiapas	3172	675	3036	926	1974	747	2275	727	279	239
Chihuahua	2549	835	2476	989	1609	675	1583	618	296	324
Mexico City	3002	408	3648	530	2608	409	2730	339	778	275
Durango	1614	1045	1571	1401	1194	1190	1345	1124	271	528
Guanajuato	5551	1083	3142	818	2511	756	2512	666	828	539
Guerrero	3427	1062	1411	654	1170	638	1226	524	211	193
Hidalgo	2124	883	2050	1126	1194	725	1147	559	144	176
Jalisco	6202	900	5272	957	3437	728	3758	687	1074	505
Mexico	8496	606	15,390	1329	6459	618	5420	469	1184	353
Michoacan	2832	691	2530	869	1511	599	1503	475	313	210
Morelos	821	504	879	719	609	547	514	359	135	237
Nayarit	935	914	666	843	780	1102	772	894	144	376
Nuevo León	1756	430	1997	520	2096	598	2354	623	490	387
Oaxaca	2407	697	2130	886	1853	888	1286	464	406	298
Puebla	4701	870	2689	678	2243	672	2572	648	440	270
Queretaro	1558	897	1411	1005	1341	1122	890	695	279	658
Quintana Roo	736	550	879	696	731	721	593	674	101	504
San Luis Potosi	2379	988	1891	1094	1511	977	1741	902	457	513
Sinaloa	3568	1368	3222	1582	2194	1163	2552	1121	566	637
Sonora	1898	775	1864	898	1901	1012	2176	982	389	514
Tabasco	3314	1563	3036	1796	2486	1725	2334	1407	364	637
Tamaulipas	1954	687	2423	968	1828	807	2018	794	389	429
Tlaxcala	623	565	772	909	731	1010	593	729	135	416
Veracruz	3682	534	3462	684	2291	477	2394	379	338	130
Yucatan	1161	619	985	650	1073	849	1128	726	296	454
Zacatecas	2152	1569	1331	1336	1146	1265	910	848	228	421
** Overall**	**79,325**	**768**	**76,766**	**933**	**53,382**	**730**	**53,389**	**623**	**11,590**	**362**
**Females**										
Aguascalientes	137	119	179	187	116	138	185	198	128	389
Baja California	467	162	512	195	256	110	463	192	120	161
Baja California Sur	165	292	128	231	47	102	130	271	38	271
Campeche	165	205	179	258	47	81	148	229	45	196
Coahuila	439	180	487	225	163	82	611	265	128	155
Colima	110	179	51	97	70	151	37	67	38	178
Chiapas	384	77	461	123	488	168	315	98	98	83
Chihuahua	824	272	384	150	465	190	407	149	271	266
Mexico City	934	126	615	83	419	59	1296	135	474	117
Durango	494	318	307	254	233	213	222	169	105	196
Guanajuato	741	134	692	154	488	128	630	147	263	148
Guerrero	604	178	384	157	116	56	352	135	211	168
Hidalgo	439	172	333	154	302	160	296	132	203	215
Jalisco	1675	241	871	148	628	123	907	148	286	115
Mexico	2197	154	1665	131	1209	105	1778	140	497	121
Michoacan	412	94	564	169	279	98	426	120	128	76
Morelos	192	114	231	165	93	72	111	69	105	157
Nayarit	165	162	179	217	186	258	93	103	75	192
Nuevo León	714	179	538	140	349	99	704	177	173	117
Oaxaca	330	88	589	205	279	114	370	117	143	89
Puebla	494	86	333	72	349	90	630	136	271	135
Queretaro	247	136	333	211	93	70	315	223	98	192
Quintana Roo	28	21	128	103	47	48	148	177	38	194
San Luis Potosi	522	210	564	287	372	215	204	97	113	118
Sinaloa	961	373	435	206	372	190	259	107	120	128
Sonora	412	175	435	212	256	137	389	172	211	255
Tabasco	494	226	461	241	442	282	407	240	143	242
Tamaulipas	494	175	922	350	233	98	574	213	211	201
Tlaxcala	192	169	102	103	93	112	93	101	45	121
Veracruz	659	93	461	78	488	89	389	56	278	93
Yucatan	275	146	179	112	163	120	259	154	60	86
Zacatecas	357	247	256	226	163	163	148	126	83	146
** Overall**	**16,721**	**158**	**13,958**	**155**	**9299**	**116**	**13,294**	**141**	**5198**	**139**

**YLL**, years of life lost; **ASYLL**, age-standardized years of life lost; **yr**., years old.

In the estimation by region (Table 3), the overall YLL in males ranged from 88.7% (Quintana Roo) to 76.1% (Baja California). The national estimate was 82.4%. The highest ASYLL rates per 100,000 inhabitants were registered in individuals from 25 to 34 years of age (526), followed by those from 15 to 24 years of age (459) and from 35 to 44 years of age (410). The overall ASYLL rate was 416 per 100,000 inhabitants and the highest state-stratified mortality rates were observed in Tabasco (851), Sinaloa (709), Durango (656), Zacatecas, (642) and Baja California Sur (570).

**Table 3.** Age-standardized years of life lost by road traffic injuries, Mexico 2014.

State		YLL, *n* (%)		% Males in YLL	ASYLL^a^					
					15–24 yr..	25–34 yr..	35–44 yr..	45–64 yr..	≥65 yr.	Overall
1	Tabasco	13,480	(4.0)	85.6	883	971	973	817	436	851
2	Sinaloa	14,250	(4.3)	84.9	874	882	668	600	375	709
3	Durango	7357	(2.2)	81.5	681	806	680	624	358	656
4	Zacatecas	6774	(2.0)	85.1	891	744	688	470	280	642
5	Baja California Sur	2606	(0.8)	80.6	555	769	411	600	418	570
6	Aguascalientes	4530	(1.4)	83.5	570	522	593	563	562	561
7	San Luis Potosi	9752	(2.9)	81.8	593	664	574	481	308	543
8	Sonora	9930	(3.0)	82.9	481	557	575	572	378	527
9	Nayarit	3993	(1.2)	82.5	539	523	677	489	283	519
10	Coahuila	10,022	(3.1)	81.8	544	695	436	462	390	517
11	Queretaro	6564	(2.1)	83.5	507	584	567	447	403	507
12	Colima	2385	(0.7)	87.2	645	640	374	436	303	500
13	Campeche	2958	(0.9)	80.3	512	695	453	471	224	497
14	Tamaulipas	11,045	(3.3)	78.0	432	652	444	495	307	483
15	Jalisco	24,110	(7.2)	81.9	569	539	414	402	295	459
16	Chihuahua	10,863	(3.2)	78.4	555	565	430	375	293	457
17	Guanajuato	17,358	(5.2)	83.8	591	460	421	389	329	449
18	Hidalgo	8234	(2.5)	80.9	517	599	423	335	197	433
19	Tlaxcala	3381	(1.0)	84.5	364	475	530	397	259	415
20	Mexico	44,294	(13.3)	83.4	378	701	349	296	225	402
21	Chiapas	12,482	(3.7)	86.0	367	498	444	408	160	397
22	Yucatan	5578	(1.7)	83.2	383	374	472	428	264	396
23	Guerrero	9113	(2.7)	81.7	608	389	330	319	180	385
24	Quintana Roo	3429	(1.0)	88.7	289	401	393	432	352	378
25	Oaxaca	9792	(2.9)	82.5	380	515	471	279	185	377
26	Puebla	14,720	(4.4)	85.9	466	351	358	373	195	367
27	Michoacan	10,497	(3.2)	82.8	383	495	333	287	139	345
28	Baja California	7621	(2.3)	76.1	336	333	359	397	188	339
29	Nuevo León	11,170	(3.4)	77.8	306	330	347	394	242	335
30	Morelos	3691	(1.1)	80.2	306	423	292	206	194	289
31	Veracruz	14,441	(4.3)	84.2	310	357	270	210	110	263
32	Mexico City	16,502	(5.0)	77.4	267	298	224	228	182	245
	**Overall**	**332,922**		**82.4**	**459**	**526**	**410**	**370**	**242**	**416**

**YLL**, years of life lost; **ASYLL**, age-standardized years of life lost; **yr**., years old.

^a^ASYLL rates per 100,000 inhabitants. The World Standard Population 2000–2025 (World Health Organization) was used.

## Discussion

Our findings suggest that the 2014 RTI burden in Mexico was 332,922 YLL and the mortality rate was higher for males (274,451 YLL, 82.4%). We identified sex, age, and region-related patterns that may be useful in designing and prioritizing intervention policies focused on the prevention of premature loss of life.

The illness burden of RTIs in Mexico was previously estimated at the national level [1] and the regional (per state) burden was not evaluated. To the best of our knowledge, this is the first study evaluating regional differences in the burden using the YLL and ASYLL rates as health measures.

The disparities between the sexes observed in our study are a common global finding and sex-related stereotypes about risk-taking and risk perception while driving have an influence on this phenomenon [17,18]. In urban areas of Mexico, male automobile drivers are more likely to be involved in alcohol-impaired driving and less likely to use seatbelts [19,20]. A higher RTI-related mortality among males has also been described in developed countries, mainly in younger individuals [21].

Nearly 75% of analyzed deaths occurred in individuals ≤44 years of age and the highest ASYLL rate (526 per 100,000 inhabitants) was observed in adults from 25 to 34 years of age. The 25–34 year age group is more likely to be employed, compared with individuals 24 years of age and younger [22], thus increasing the economic burden.

Heterogeneous state-specific ASYLL rates were found. The highest rate was observed in Tabasco (southeastern region of Mexico), where 5.3% of all deaths registered in 2014 were due to RTIs. This is double the number of the national estimate (2.5%) within the same period. Interestingly, the motorization rate of Tabasco is lower than the average rate (273.1 vs. 310.3 per 100,000 inhabitants, respectively), but the number of accidents occurring on federal highways (17.6%) and the total number of deaths involving motorcycle riders (36.5%) were higher than the national estimates (5.4% and 12.4%, respectively). These events may have determined the scenario observed.

The overall ASYLL rates were low in wealthy (i.e. Nuevo León) and highly motorized (i.e. Mexico City) states, perhaps due to the quality of emergency care provided and the exposure of populations particularly vulnerable to traffic [23]. Within-country disparities in RTI burden have been described in other populations [21,24,25]. This fact may be secondary to multiple risk exposures, including infrastructural characteristics, health care facilities and alcohol drinking prevalence [25].

The conceptual framework of traffic injuries is complex. They result from the interaction of road users, vehicles, and infrastructure [26]. The published data regarding interventions to reduce the related burden in low- and middle-income countries is limited [27]. The World Report on RTI prevention highlights the need for an integrated effort focusing on improved information systems, response capacity strengthening, and reduced exposure to modifiable risk factors plus the availability of resources for targeting them [17].

Several risks factors associated with increased RTI risk and death have been documented in the Mexican population. These factors include those influencing the occurrence of the crash (speeding, alcohol consumption, and hand-held mobile phones) and injury severity (non-use of crash helmets by two-wheeled vehicle users and non-use of retention devices, such as seat belts and child safety seats) [28–30].

Most of the cost-effectiveness strategies to reduce the RTI burden in low- and middle-income countries are linked to legislative interventions [27]. The current Mexican road legislation is permissive and poorly applied [31]. An integrated juridical effort is needed that includes the regulation of alcohol-impaired driving, prohibition of talking on hand-held phones while driving, verification of the use of retention devices by drivers and occupants, and high penalties for offenders. Susceptible populations must be included [32].

The population mobility patterns have changed and a constant growth in motorcycle users has been observed since 2002 [33]. A simultaneous increase in fatal motorcycle injuries has been documented [6]. In addition, also in Mexico, high mortality rates secondary to intentional injuries (i.e. homicide and suicide) are observed [34].

The potential limitations of our study must be cited. First of all, despite the fact that official mortality data were used to compute the YLL and ASYLL rates, the burden of disease may be underestimated, given that approximately 30% of fatal RTIs are misclassified [35]. Second, data to estimate the RTI-associated YLD are not systematically collected in Mexico and therefore we were unable to compute the DALY. However, the YLL and ASYLL rates are valid stand-alone indicators for quantifying premature mortality due to specific events [36–38]. Third, a clustered analysis was performed and no specific populations involved in those injuries were identified. Governmental data indicate that the highest number of victims of fatal RTIs in 2013 were pedestrians (51.5%), followed by occupants of four-wheeled automobiles (34.4%), motorcycle riders (12.3%), and bicyclists (1.8%) [39].

## Conclusions

Our findings provide quantitative evidence of the burden of RTIs in Mexico. These are preventable events and efforts to reduce the associated economic and social burden must be made. Sex, age, and region-related patterns were highlighted in our study and they may be useful in improving the impact of public policies focused on the prevention of premature loss of life secondary to traffic injuries.

## Paper context

The road traffic injuries-related mortality in Mexico has been evaluated using traditional rates that fail to highlight the premature deaths. The years of life lost due to these injuries during 2014 were estimated in this study and sex, age, and region-related differences were evaluated. The obtained estimators may useful to improve the impact of public policies focused on the prevention of these events.
